# Common artefacts encountered on images acquired with combined compressed sensing and SENSE

**DOI:** 10.1007/s13244-018-0668-4

**Published:** 2018-11-08

**Authors:** Thomas Sartoretti, Carolin Reischauer, Elisabeth Sartoretti, Christoph Binkert, Arash Najafi, Sabine Sartoretti-Schefer

**Affiliations:** Institut für Radiologie, Kantonsspital Winterthur, Brauerstrasse 15, 8401 Winterthur, Switzerland

**Keywords:** Artefacts, Magnetic resonance imaging, Motion artefacts, Compressed SENSE technique, Scan time reduction

## Abstract

**Abstract:**

Various techniques have been proposed which aim at scan time reduction and/or at improved image quality by increasing the spatial resolution. Compressed sensing (CS) takes advantage of the fact that MR images are usually sparse in some transform domains and recovers this sparse representation from undersampled data. CS may be combined with parallel imaging such as sensitivity encoding (SENSE), hereafter referred to as Compressed SENSE, to further accelerate image acquisition since both techniques rely on different ancillary information. In practice, Compressed SENSE may reduce scan times of two-dimensional (2D) and three-dimensional (3D) scans by up to 50% depending on the sequence acquired and it works on 1.5-T or 3-T scanners. Compressed SENSE may be applied to 2D and 3D sequences in various anatomies and image contrasts. Image artefacts (i.e. motion, metal and flow artefacts, susceptibility artefacts) frequently appear on magnetic resonance images. The Compressed SENSE technique may cause special artefacts, which might influence image assessment if they go undetected by imaging readers. Our institution has been using Compressed SENSE for over half a year, both in a neuroradiological setting and for musculoskeletal examinations. So far, three special image artefacts—called the wax-layer artefact, the streaky-linear artefact and the starry-sky artefact—have been encountered and we aim to review these main artefacts appearing in sequences acquired with Compressed SENSE.

**Teaching Points:**

*• Compressed SENSE combines compressed sensing and SENSE technique.*

*• Compressed SENSE permits scan time reduction and increases spatial image resolution.*

*• Images acquired with Compressed SENSE may present with special artefacts.*

*• Knowledge of artefacts is necessary for reliable image assessment.*

## Introduction and technical part

Magnetic resonance imaging (MRI) data are collected in the k-space which is mathematically related to image space through the Fourier transformation [1]. Collecting all points in the k-space is time-consuming [1]. Both parallel imaging such as sensitivity encoding (SENSE) and compressed sensing (CS) are techniques that permit reducing the amount of acquired k-space data, thus enabling an acceleration of MRI acquisitions. Using SENSE, the spatial sensitivities of each receiver in a multi-coil array are used to reconstruct the MR image from undersampled k-space data [2]. Alternatively, CS exploits sparsity in MR images to recover MR images from undersampled k-space data [3]. Some images, such as angiograms, are already sparse in their pixel representation, while others have a sparse representation in an appropriate transform domain, such as the Wavelet domain [3].

Both methods, namely compressed sensing and SENSE, rely on different ancillary information and may thus be combined to further reduce scan time [4], a technique hereafter referred to as Compressed SENSE by Philips Healthcare [5]. In the Philips implementation of Compressed SENSE, a variable density subsampling scheme is combined with an iterative reconstruction algorithm that allows the combination of wavelet transformation of compressed sensing with coil information of SENSE, hence the name Compressed SENSE [5]. The denoising level that can be chosen in this technique as weak, medium or strong, gives a balance between data consistency and sparsity constraining and thus allows the user to select a preferred image appearance. Images reconstructed with a weak denoising level have a noisier appearance than images reconstructed with medium or strong level [5]. No additional filter is used in our institution.

Undersampling in general can be performed randomly, radially or by a variable density incoherent scheme, where more data are sampled in the centre of the k-space than in the periphery [1, 5]. In all these non-uniform data sampling methods, noise-like incoherent artefacts distributed in a non-uniform pattern on the image may occur [5], leading to noise-enhancement with noisy and blurred images and to semi-circular ringing artefacts [1, 6]. Radial undersampling additionally may lead to streaky artefacts, whereby the main body of the image is still recognisable, as the centre of the k-space is well sampled [1]. Thus, the undersampling method performed dictates the kind of artefacts that may occur in the final image.

Usually, a sparse reconstruction technique is used to reconstruct full images from undersampled data [1], which also allows for elimination of these artefacts. The image generated must be consistent with the data collected [1].

CS utilises the non-uniform variable density incoherent and sparse undersampling scheme, resulting in artefacts distributed incoherently over the image, while SENSE depends on a uniform undersampling scheme, resulting in regular, back-folding artefacts [5].

Compressed SENSE is field-strength independent; it may be applied to nearly all image contrasts with Cartesian acquisition schemes in all anatomies with 2D and 3D sequences (excluding EPI scans as in diffusion weighted imaging, diffusion tensor imaging, perfusion MRI with dynamic susceptibility weighted imaging or in resting state fMRI) [5].

Image artefacts that stem from Compressed SENSE should be known by radiologists [1] as they disturb the image impression of the reader [7] and possibly might prevent a correct diagnosis.

Previous studies have only investigated CS artefacts on very few sequences and the CS technology utilised in previous studies is different to the Compressed SENSE technology used by Philips Healthcare. We present special image artefacts occurring in Compressed SENSE technique combining compressed sensing and SENSE in neuroradiological and musculoskeletal MRI examinations based on our 6 months` experience with this technique.

## Materials and methods

The Compressed SENSE factor determines the acceleration of the sequence and thus the scan duration. The Compressed SENSE factor and the denoising level (weak, medium, strong) influence the quality of image. The denoising level establishes an equilibrium between the undersampling of data and their necessary consistency in order to reconstruct a reliable image. A weak denoising level leads to a noisier image impression than a medium or strong denoising level [5].

In the present work, Compressed SENSE factors and denoising levels applied were determined in sequences obtained in volunteers and these Compressed Sense factors and denoising levels are depicted in Table 1.

**Table 1:** Compressed SENSE factors and denoising levels for 1.5-T and 3-T MR examinations

Sequence	CS SENSE factor	Denoising level	Scan time reduction
3D FLAIR	8.2	medium	30%
3D DIR	6.7	medium	30%
3D m-Dixon T1 TFE	7	medium	35%
3D T1 TFE	2.5	weak	50%
3D T1 black blood TSE	5.75	weak	25%
SWIP	5.7	weak	40%
3D TOF MRA	3	strong	40%
3D PCA	4	strong	50%
3D T2 DRIVE TSE	2.9	strong	30%
3D T1 spine view	5.2	strong	only with Compressed SENSE
3D T2 spine view	7	strong	only with Compressed SENSE
3D PD knee	7.5	strong	only with Compressed SENSE

*FLAIR* fluid attenuated inversion recovery, *DIR* double inversion recovery, *m-Dixon* modified Dixon, *TFE* turbo field echo, *TSE* turbo spin echo, *SWIP* susceptibility-weighted imaging phase, *TOF MRA* time-of-flight magnetic resonance angiography, *PCA* phase-contrast angiography, *PD* proton density

Twenty volunteers, nine women and 11 men, mean age of 28.95 years (range, 19–37 years; SD, 4.72 years) were scanned with varying Compressed SENSE factors and denoising levels and the acquired images of each sequence were examined by three radiologists/neuroradiologists, who then chose the images with the highest possible Compressed SENSE factor and denoising level with the identical image impression as the original sequence without Compressed SENSE. Only young healthy volunteers without any neurological and musculoskeletal disease were chosen, as volunteers were required to remain as still as possible during the examination. Volunteers were examined both on a 1.5-T Philips Ingenia MR scanner and on a 3-T Philips Achieva scanner.

In 4 months, a total of 850 brain MRI examinations (291 at 1.5 T and 559 at 3 T), 169 knee (138 at 1.5 T and 31 at 3 T) and 263 lumbar and cervical spine examinations (219 at 1.5 T and 44 at 3 T) were performed in patients during the daily clinical routine. Imaging artefacts were then analysed by two radiologists: a senior neuroradiologist with 28 years of experience and a chief resident with 4 years of experience. The type of imaging artefacts in different MR sequences was analysed in the volunteer studies and the frequency of these artefacts in different sequences was determined in the clinical patient examinations. Furthermore, in 14 tests with three volunteers (T.S. 19 years, E.S. 21 years and S.S. 54 years), possible inherent differences between motion artefacts on images with or without Compressed SENSE were analysed. The volunteers were scanned while being instructed not to move their heads or eyes or to actively move the head (from left to right or to nod) or the eyes (eye rolling) at a specific rate of about 80 movements per minute (slight movements of a few millimetres or severe movements of the head of 2–3 cm) during a set time period. Two-dimensional T2-weighted turbo spin echo (TSE) transverse images were acquired with different phase directions (anterior/posterior or left/right). The sequences were acquired with identical parameters both with and without Compressed SENSE.

Images presented in this work were obtained in the volunteers and in three patients. Written consent was obtained from all volunteers as well as from three patients who agreed to publication of selected images. Further ethical committee approval was waived for all other patients as only the frequency of artefacts was determined within the scope of internal quality control process.

## Results

The following artefacts did occur on images acquired with the Compressed SENSE technique both in volunteers (without contrast administered) and in-patient examinations (with and without contrast administered).

### Motion artefacts (Fig. 1)

**Fig. 1:**
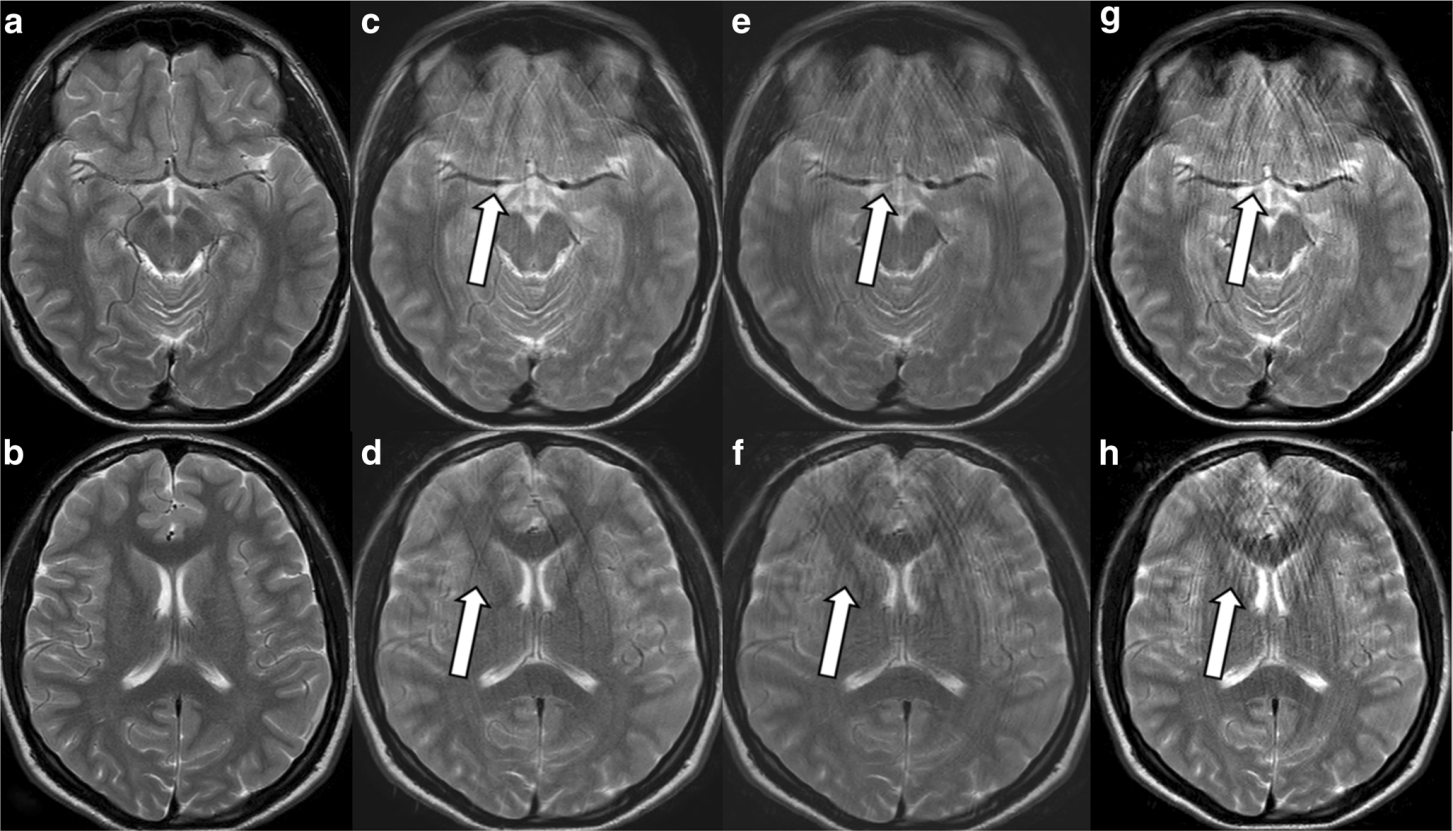
Motion artefacts on images acquired with or without CS SENSE. A transverse 2D T2 TSE sequence was acquired; without Compressed SENSE (**a**-**d**) and with Compressed SENSE Factor 2 in Fig. 1e, h. In **a** and **b**, the volunteer did not move. In **c** and **d**, slight head movements from left to right were performed. Motion artefacts visible as semi-circular rings in phase direction left-right were obtained. In **e** and **f**, slight head movements from left to right and, in **g** and **h**, severe head movements were performed during scanning. The frequency of the motion artefacts visible as semi-circular rings in phase direction left-right was considerably increased from slight to severe head movements

In all volunteer tests, motion artefacts appeared in phase direction as semi-circular rings on images acquired without compressed SENSE (Fig. 1c, d) or with compressed SENSE (Fig. 1e-h) after slight (Fig. 1d, e) or severe (Fig. 1g, h) head movements from left to right if compared to images obtained without head movement (Fig. 1a, b). The artefact appeared with higher spatial frequency if head movement was increased and if Compressed SENSE was applied (Fig. 1g, h compared to Fig. 1e, f). The visual appearance of the motion artefact itself was indifferent to the use of Compressed SENSE or to the Compressed SENSE factor chosen (Fig. 1c-f). Head movement can easily be identified as blurring of the background outside the skull, when applying different window and level settings.

### Special artefacts related to the Compressed SENSE technique

These artefacts were only present in sequences acquired with Compressed SENSE and could be seen on images with or without additional motion artefacts. Artefacts were enhanced by patient motion. Artefacts looked identical on sequences obtained on 1.5-T and 3-T MR scanners.

#### The wax-layer artefact (Fig. 2)

**Fig. 2:**
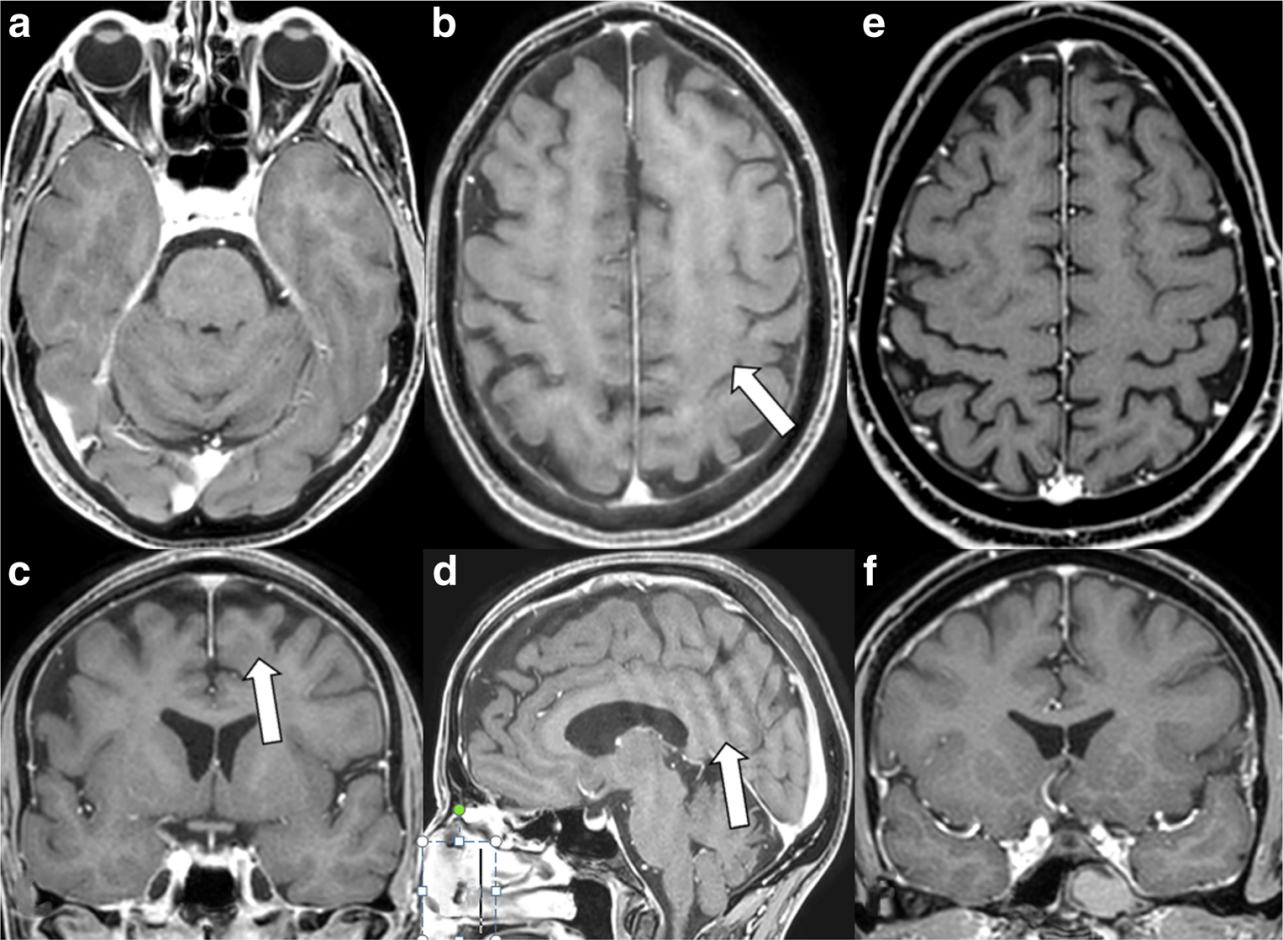
The wax-layer artefact. On reconstructed transverse postcontrast 3D T1 m-Dixon TFE image obtained with Compressed SENSE the more cranial parts of the brain showed an inhomogeneous appearance as if the image was covered by a wax layer (**a** and **b**, *white arrow*). This artefact presented as low frequency ringing artefact on coronal reconstructed images (**c**, *white arrow*) and on sagittal source images (**d**, *white arrow*). For comparison: this artefact was never detected on postcontrast 3D T1 m-DIXON TFE acquired without Compressed SENSE (**e** and **f**)

This artefact only appeared on the 3D T1 m-Dixon turbo field echo (TFE) sequence of brain studies acquired with Compressed SENSE (postcontrast 3D T1 m-Dixon TFE as shown in Fig. 2a-d) but not on corresponding images of the same sequence without Compressed SENSE (Fig. 2e, f). The MR images appeared to be covered in a thin inhomogeneous layer of wax, thus leaving the impression of a slight turbidity on the anatomical structures depicted. This artefact was visible on transverse reconstructions (Fig. 2a, b). It was more prominent in the cranial parts of the brain (Fig. 2b) where the differentiation of grey and white matter was already reduced due to inhomogeneity of the B_1_-field. On coronal reconstructions and on sagittal source images, the artefact presented as a low frequency ringing artefact (Fig. 2c, d) instead of the wax layer artefact. We suspected that this artefact might partially be caused by inhomogeneity in the B_1_-field and by the undersampling technique used [1].

#### The streaky-linear artefact

There were three types of streaky-linear artefacts crossing the image in an oblique diagonal or horizontal manner in brain and knee studies. Type A and B streaks were visible on brain examinations and type C streaks only in knee examinations.

**Fig. 3:**
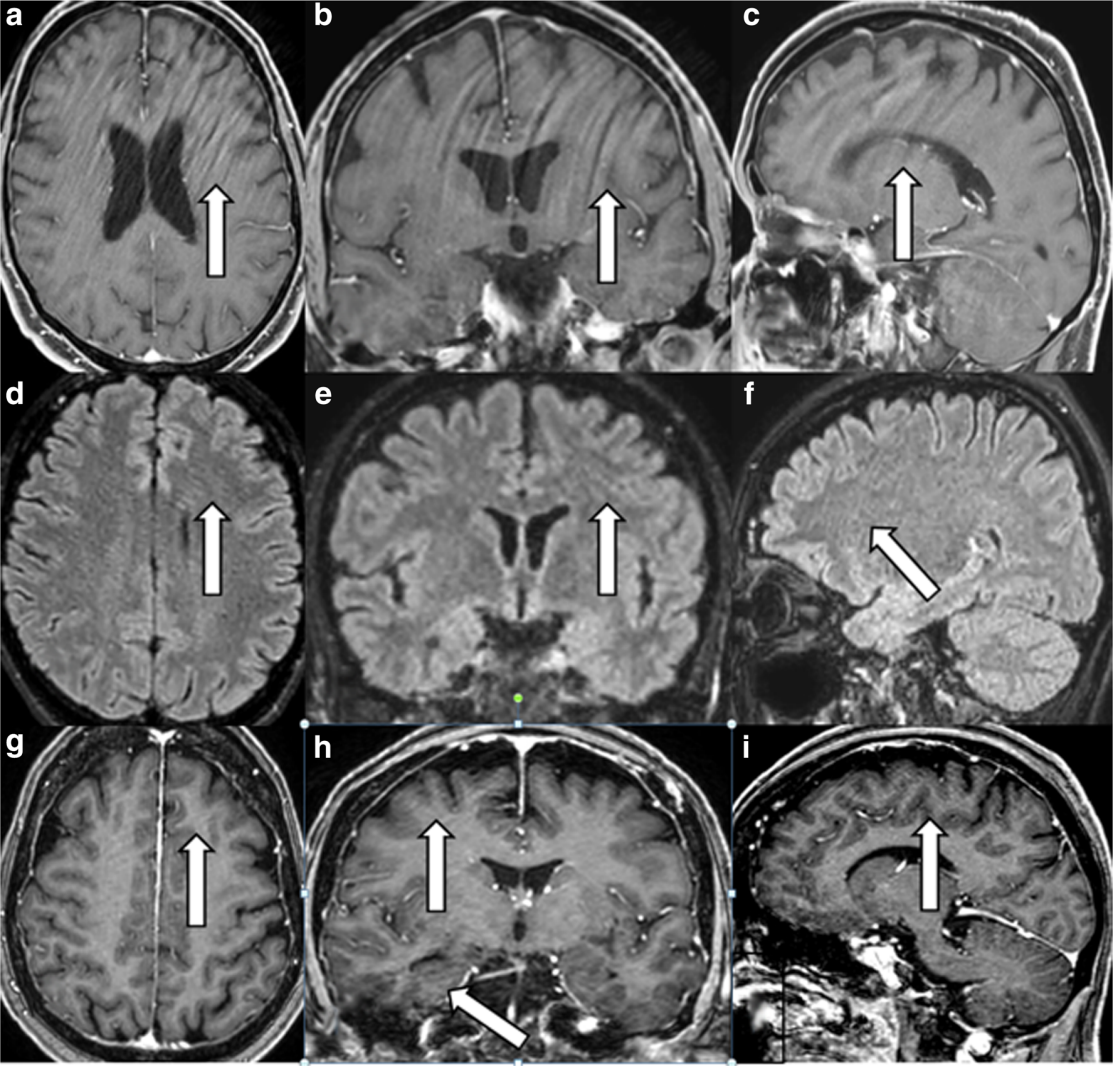
The streaky linear artefact type A. Streaky artefacts type A (*white arrows*) on postcontrast 3D T1 m-Dixon TFE (F**a**-**c**) and on 3D FLAIR (**d**-**f**) and on 3D T1 TFE postcontrast sequence (**g**-**i**). The streaks are horizontal or oblique, long or short lines, most obvious on reconstructed transverse images (**a**, **d**, **g**) and on reconstructed coronal images (**b**, **e**, **i**). The streaks are less obvious infratentorially or on sagittal source images (**c**, **f**, **i**) where the streaks appear as slightly curved ringing artefacts in various spatial frequencies

Type A streaks, either long or short lines as demonstrated in Fig. 3a–i, appeared centrally and peripherally on the image in a horizontal or oblique arrangement. They were often more prominent on transverse (Fig. 3a, d, g) and on coronal image reconstructions (Fig. 3b, e, h), but less prominent in infratentorial regions (Fig. 3c, f, i) and on sagittally scanned images (Fig. 3c, f, i).

Type-A streaks could always be provoked on transverse, coronal and sagittal images if the reconstruction voxel (0.5 mm) was smaller than the acquisition voxel (1 mm) as seen on the postcontrast 3D T1 TFE sequence acquired with Compressed SENSE (Fig. 3g–i).

Type-A streaks appeared on the precontrast and postcontrast images of 3D m-Dixon T1 TFE (Fig. 3a-c) or on the 3D FLAIR sequence (Fig. 3d–f), but only rarely on the 3D DIR, the 3D T1 black blood TSE or the 3D T1 FFE sequence as depicted on Table 2.

**Table 2:** Frequency of unique Compressed SENSE artefacts on various sequences

Sequence	Total no. of cases	Without artefact	Wax-layer artefact	Streaky-linear type-A artefact	Streaky-linear type-B artefact	Starry-sky artefact
3D FLAIR	739	540 (73%)	0	44 (6%)	44 (6%)	111(15%)
3D m-Dixon TFE	425	336 (79%)	34 (8%)	30 (7%)	0	25 (6%)
3D T1 black blood TSE	272	267 (98%)	0	5 (2%)	0	0
3D DIR	161	151 (94%)	0	0	5 (3%)	5 (3%)
3D T1 TFE	425	391 (92%)	0 (0%)	8 (2%)	0	26 (6%)

**Fig. 4:**
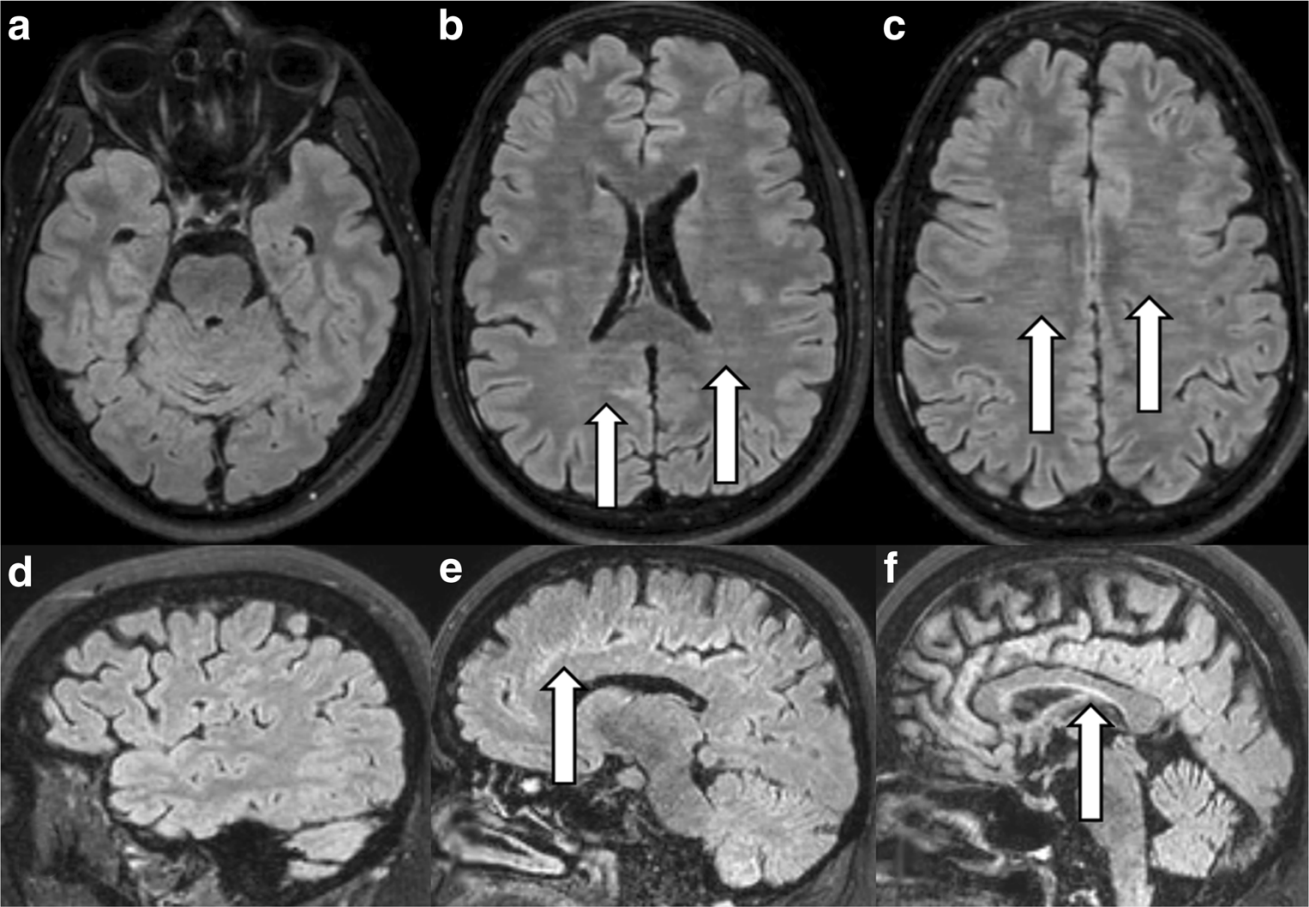
The streaky-linear artefact type B. Streaky-linear artefacts type B (*white arrows*) are horizontally oriented lines only in the centre of the transverse reconstructed image on 3D FLAIR (**b**, **c**) that are not visible or less visible along the image periphery (**d**) or infratentorially (**a**). There are faintly oblique lines on sagittal source images (**e**, **f**)

Type-B streaks were best seen centrally within the transverse image reconstruction on the precontrast and postcontrast 3D FLAIR images (Fig. 4g, h) and rarely on 3D DIR (Table 2). The streaks were only horizontally arranged (Fig. 4g, h) and occurred less often in the image periphery along the cortex (Fig. 4g, h) and in the infratentorial regions (Fig. 4g, i). There were faint oblique or crossing oblique lines on sagittally scanned images (Fig. 4i).

**Fig. 5:**
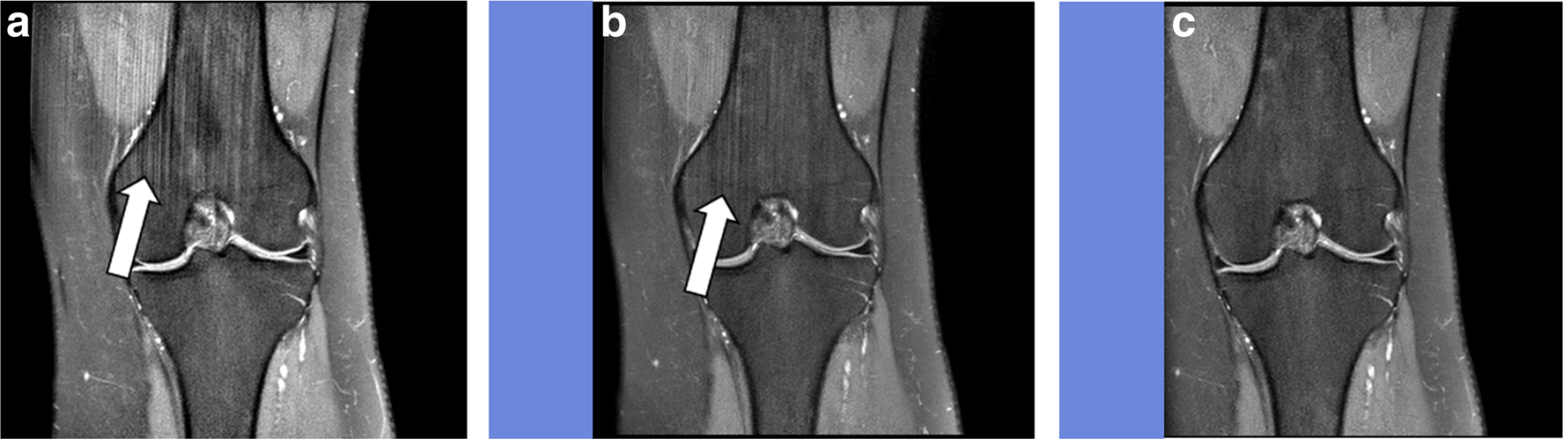
Streaky-linear artefact type C. Coronal reconstructed 3D PD sequence of the knee with large FOV and without rest slab (**a**). The streaky-linear artefacts are depicted on the coronal reconstructed image (*white arrow*). If a rest slab (*blue band*) is placed with a small gap near to the scanned object (i.e. knee) the amount of artefacts is reduced (*white arrow* in **b**). If the rest slab is placed directly adjacent to the scanned object (i.e. knee) without any gap as shown in **c** and an oversampling rate of 10% is chosen, the artefact completely disappears

Type-C streaks only appeared on the coronal reconstructions of the sagittally scanned 3D PD SPAIR sequence of the knee (Fig. 5), when the field of view (FOV) was too small for the object imaged. This artefact was an aliasing artefact, also called fold-over artefact, with vertical lines in high frequency over the image. Usually, fold-over artefacts are visible in all imaging planes [8], but this was not true for the 3D PD SPAIR sequence acquired with Compressed SENSE. To avoid this artefact, a rest slab had to be placed directly adjacent to the object scanned without any gap (Fig. 5c) and an oversampling rate of 10% had to be added.

Type-A and -B streaky artefacts are not fold-over artefacts, as the FOV was always large enough.

#### The starry-sky artefact (Fig. 6)

**Fig. 6:**
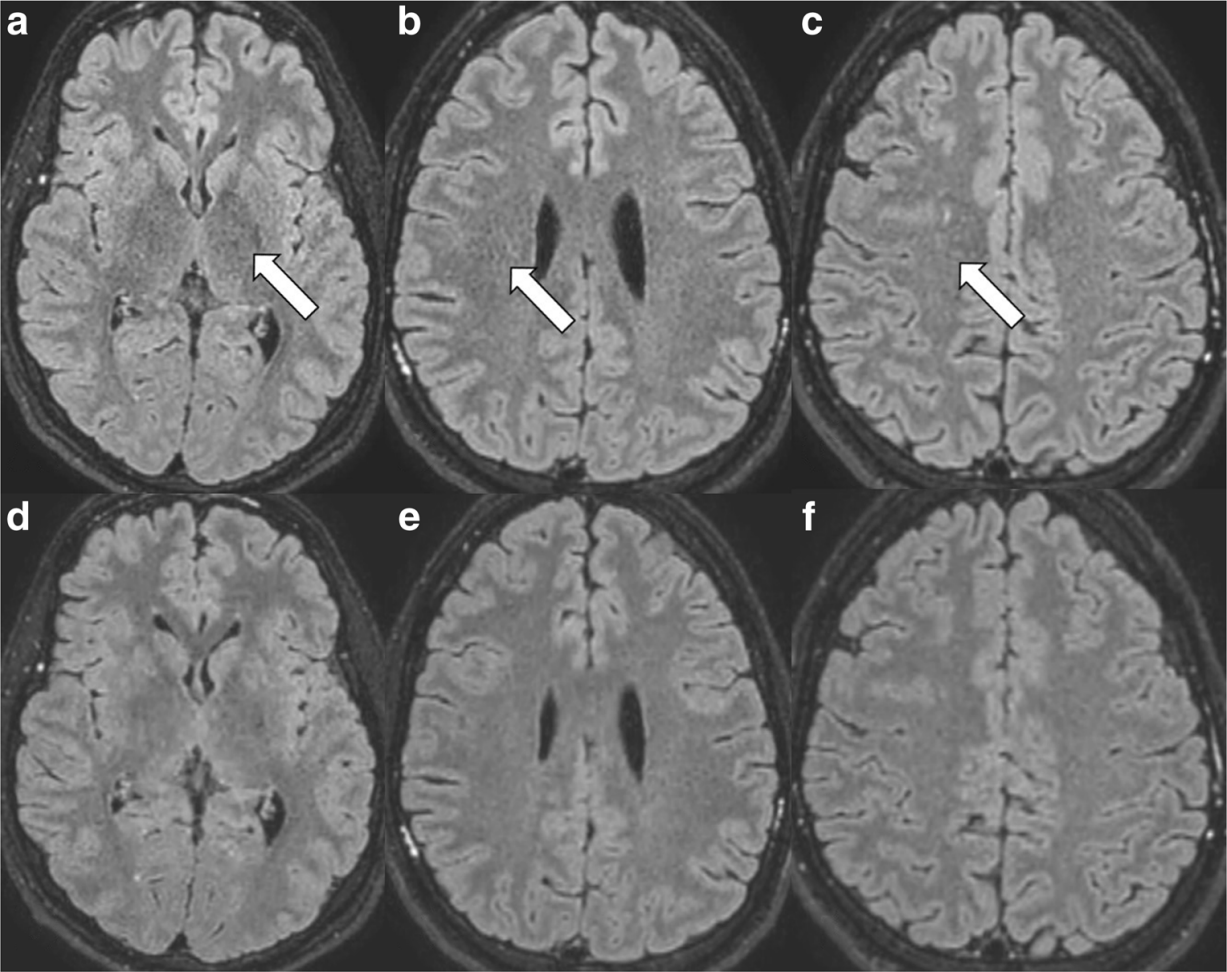
The starry-sky artefact. This artefact was visible as grainy image noise (*white arrows*) on the transverse reconstructions of the 3D FLAIR sequence with Compressed SENSE (**a**-**c**) if compared to corresponding transverse reconstructed images of a 3D FLAIR with Compressed SENSE but without this artefact in the same volunteer (**d**-**f**) after sequence repetition. The artefact was also visible on coronal reconstructions and on sagittal source images (not depicted) of the 3D FLAIR sequence

Structures affected by this artefact seemed slightly pixelated and grainy. Small circular (Fig. 6a-c) and sometimes also small semi-circular rings and streaks (more frequently along the brain periphery than in the centre) occurred in combination with this artefact. The image impression reminded us of a night sky with millions of stars glowing. The frequency of this artefact is depicted in Table 2. The image impression was similar to the noise enhancement/image blurring as described in CS [1, 7, 9]. The artefact was pronounced in the centre of the image.

It occurred on precontrast and postcontrast images of the 3D FLAIR, 3D m-Dixon T1 TFE, 3D DIR and the 3D T1 TFE sequence.

In general unique Compressed SENSE related artefacts were much more common with 3D FLAIR and with 3D T1 m-Dixon TFE than with 3D T1 TFE, 3D DIR and with 3D T1 black blood TSE (*see* Table 2). Artefacts were never present on 3D T2 DRIVE, 3D TOF, 3D PCA, 3D T1 spine view or on 3D T2 spine view sequence.

Strategies for artefact correction related to the different image artefacts are depicted in Table 3.

**Table 3:** Strategies for correction of different artefact types

Type of artefact	Strategy for artefact correction
Type-A streaks	The reconstruction voxel must be at least the same size as the acquisition voxel
Type-B streaks	Repetition of sequence
Type-C streaks	a rest slab has to be placed directly adjacent to the object scanned without any gap and an oversampling rate of 10% has to be added
Wax-layer artefact	Repetition of sequence
Starry-sky artefact	Repetition of sequence

## Discussion

In general MR images obtained with Compressed SENSE were not more susceptible to motion artefacts than MR images obtained without Compressed SENSE technique as could be demonstrated in our volunteer experiments. But motion artefacts, visible as semi-circular rings in phase direction, were depicted in higher spatial frequency on images acquired with Compressed SENSE as seen in Fig. 1e–h. However, inherent special artefacts, strongly aggravated by patient movements, namely the wax layer artefact, the streaky-linear artefact and the starry sky artefact were present on our routine clinical sequences 3D fluid-attenuated inversion recovery (FLAIR), 3D T1 modified (m)-Dixon TFE, more rarely on 3D T1 TFE, 3D double inversion recovery (DIR) or 3D T1 black blood TSE of brain examinations and on 3D proton density (PD) spectral attenuated inversion recovery (SPAIR) of knee examinations if these sequences were acquired by Compressed SENSE technique. However, these artefacts were never encountered on sequences acquired with Compressed SENSE technique and with high contrast to noise such as 3D T2 driven equilibrium (DRIVE), 3D time of flight (TOF) or 3D phase contrast angiography (PCA) and 3D T1 spine view or 3D T2 spine view.

To our knowledge, these special artefacts are a finding not yet reported in Compressed SENSE technique. Artefacts appeared in sequences obtained with and without contrast agent administered and thus were independent of a possible contrast agent administration. Common artefacts encountered with echo planar imaging (EPI) techniques, such as chemical shift artefacts in the phase encoding direction, susceptibility artefacts, EPI ghosting artefacts, EPI distortions and saturation arfefacts [8], are not present in sequences obtained with Compressed SENSE as Compressed SENSE is not compatible with the EPI technique.

Radiologists must be aware of these special Compressed SENSE-related artefacts in order to make correct MRI-based radiological diagnoses. According to our personal experience the artefacts, especially the starry-sky artefacts and the wax-layer artefacts, were only slightly disturbing the image quality and did not affect the image assessment. Streaky artefacts were even less disturbing because of their regular appearance as already described in the literature [1, 7, 9]. Most probably, the unique artefacts encountered using Compressed SENSE were caused by a combination of artefacts already known from the SENSE technique (ghosting artefacts) as well as from the CS technique.

Artefacts caused by CS are unpredictable, often not easily recognisable and of unfamiliar nature. Furthermore, they often appear with predilection in certain parts of the images or in selected image planes thus resulting in noise enhancement and global ringing [1, 7, 9].

The different undersampling techniques (regular, radial and random) as described in SENSE technology and in CS theory are firstly known to provoke fold-over artefacts (in regular undersampling) and linear artefacts (in random and radial undersampling) corresponding to streaky-linear artefacts in Compressed SENSE, are secondly known to provoke noise enhancement, corresponding to starry-sky artefact in Compressed SENSE and are thirdly known for provoking global ringing, corresponding to wax-layer artefact and streaky-linear artefacts type A in Compressed SENSE.

Two other mechanisms are known to cause unique Compressed SENSE-related artefacts as well. First, if the reconstruction voxel is smaller than the acquisition voxel, streaky linear artefacts type A occur as demonstrated in Fig. 3g-i. Second, if the knee is scanned without a directly adjacent rest slab and without oversampling, then streaky-linear artefacts type C are produced as shown in Fig. 5. These artefacts can be avoided if the reconstruction voxel has at least the same size as the acquisition voxel for type-A streaky-linear artefacts and if a directly adjacent rest slab is added together with a 10% oversampling rate in type C streaky-linear artefacts.

There are possible limitations to our results:

First, the frequency of special artefacts in different sequences was not separately evaluated for examinations obtained on 1.5-T and on 3-T machines. However, the frequency of artefacts might be influenced by the field strength.

Second, the presence of artefacts was not evaluated in examinations of heart, abdomen and pelvis.

Third, the Compressed SENSE technique is specifically designed for Philips scanners. Other vendors present acceleration techniques called “HyperSense” in GE scanners and “Turbo Suite” in Siemens scanners as well. However, we do not know if these acceleration techniques are fully comparable in their technical background. Most importantly the artefacts are heavily dependent on the background of the acceleration technique used. Thus, our results are not fully applicable to images acquired with techniques from other vendors than Philips Healthcare. We recommend to perform an additional study that analyses artefacts in the different acceleration techniques obtained by the different vendors.

Fourth, while all artefacts described were validated both in volunteers and in patients, we did not administer contrast agent to volunteers due to ethical considerations.

Sixth, we do not know if the artefacts related to the Compressed Sense technique might also be present on images obtained with non-compressed sensing reconstructions and further tests are necessary to compare artefacts related to Compressed SENSE technique with artefacts in non-compressed sensing acquisitions.

Seventh, artefacts were especially encountered on sequences with low contrast to noise, possibly resulting from undersampling in the k-space in these sequences. However, no tests were performed whether these artefacts could possibly disappear if the contrast to noise in these sequences was increased.

In conclusion, compressed SENSE combines the CS and the SENSE technique. Compressed SENSE permits scan time reduction and an increase in spatial image resolution. However, images acquired with compressed SENSE may depict special imaging artefacts, namely the wax-layer, the streaky-linear and the starry-sky artefacts that can be especially encountered on sequences with low contrast to noise possibly resulting from undersampling in the k-space. They are often more obvious on selected image regions and planes and are greatly enhanced by patient movements.

Sequences with high contrast to noise do not present with any artefacts if scanned with Compressed SENSE technique.

The radiologist must be aware of these artefacts for reliable and unaffected image interpretation and assessment.

## Abbreviations

CS: Compressed sensing
